# Areas of patent innovation regarding diabetic foot ulcers: technological prospecting

**DOI:** 10.17533/udea.iee.v44n1e08

**Published:** 2026-03-29

**Authors:** Vanessa Faria de Freitas, Marcos Vinicius Silva Mendes, Sarah Ferreira Neves, Rosana Maria Barreto Colichi, Juliano Teixeira Moraes, Helen Cristiny Teodoro Couto Ribeiro

**Affiliations:** 1 Nurse student. Email: vanessafaria33@gmail.com https://orcid.org/0000-0002-6191-3415 Universidade Federal de São João del-Rei Brazil vanessafaria33@gmail.com; 2 Nurse student. Email: marcosvinicius5280@gmail.com https://orcid.org/0000-0002-7156-6234 Universidade Federal de São João del-Rei Brazil marcosvinicius5280@gmail.com; 3 Nurse student. Email: sarahferreira279@gmail.com https://orcid.org/0000-0002-7104-9149 Universidade Federal de São João del-Rei Brazil sarahferreira279@gmail.com; 4 Nursing researcher, Ph.D. Email: rosana.barreto-colichi@unesp.br https://orcid.org/0000-0002-8765-3965 Universidade Estadual Paulista Brazil rosana.barreto-colichi@unesp.br; 5 Nurse, Ph.D. Assistant Professor. Email: julianotmoraes@ufsj.edu.br https://orcid.org/0000-0002-1109-962X Universidade Federal de São João del-Rei Brazil julianotmoraes@ufsj.edu.br; 6 Nurse, Ph.D. Assistant Professor. Email: helen.cristiny@ufsj.edu.br. Corresponding author. https://orcid.org/0000-0001-9365-7228 Universidade Federal de São João del-Rei Brazil helen.cristiny@ufsj.edu.br; 7 Federal University of São João del-Rei (UFSJ), Divinópólis, Brazil Universidade Federal de São João del-Rei Federal University of São João del-Rei (UFSJ) Divinópólis Brazil; 8 São Paulo State University (UNESP), Medical School, Botucatu, Brazil Universidade Estadual Paulista São Paulo State University (UNESP) Medical School Botucatu Brazil

**Keywords:** diabetic foot, inventions, diabetes mellitus, factual databases, record system, patent., pie diabético, invenciones, diabetes mellitus, bases de datos factuales, sistema de registros, patent., pé diabético, invenções, diabetes mellitus, bases de dados factuais, sistema de registros, patente.

## Abstract

Abstract

**Objective.:**

To identify areas of innovation in internationally registered patents focused on diabetic foot ulcers**.**

**Methods.:**

This is a quantitative technological prospecting of international patents, from 2020 to 2024, in the World Intellectual Property Organization database, using the descriptor "diabetic foot".

**Results.:**

1372 patents were found, with the largest number of registrations originating from China (76.38%), the United States (4.74%), the Patent Cooperation Treaty (3.8%), and India (3.1%). The areas of innovation of the patents were classified into treatment (82.1%), prevention (12.2%), and evaluation (5.7%). Among the patents, 72.8% correspond to medical devices and 27.2% to pharmaceutical compounds**.**

**Conclusion.:**

The global trend is towards patents for the treatment of diabetic foot complications.

## Resumen

**Objetivo.:**

Identificar las áreas de innovación de las patentes registradas internacionalmente relacionadas con la úlcera del pie relacionada con la diabetes**.**

**Métodos.:**

Se trata de una prospección tecnológica cuantitativa de patentes internacionales, en un período de 2020 a 2024. Se utilizó la base de datos de la Organización Mundial de la Propiedad Intelectual, utilizando el descriptor «diabetic foot» (pie diabético).

**Resultados.:**

Se encontraron 1372 patentes, con el mayor número de registros procedentes de China (76.38 %), Estados Unidos (4.74%), el Tratado de Cooperación en materia de Patentes (3.8%) y de la India (3.1%). Las áreas de innovación de las patentes se clasificaron en tratamiento (82.1%), prevención (12.2%) y evaluación (5.7%). Dentro de las patentes, el 72.8 % corresponde a dispositivos médicos y el 27.2 % a compuestos farmacéuticos.

**Conclusión.:**

La tendencia mundial se centra en las patentes para el tratamiento de complicaciones del pie relacionadas con la diabetes.

## Resumo

**Objetivo.:**

Identificar as áreas de inovação de patentes registradas internacionalmente voltadas à úlcera no pé relacionada ao diabetes.

**Métodos.:**

Trata-se de prospecção tecnológica quantitativa de patentes internacionais, no periodo de 2020 a 2024, na base de dados da *World Intellectual Property Organization*, utilizando o descritor “*diabetic foot”*.

**Resultados.:**

foram encontradas 1372 patentes, com maior número de registros provenientes da China (76.38%), Estados Unidos (4.74%), Patent Cooperation Treaty (3.8%) e Índia (3.1%). As áreas de inovação das patentes foram classificadas em tratamento (82.1%), prevenção (12.2%) e avaliação (5.7%). Dentre as patentes, 72.8% correspondem a dispositivos médicos e 27.2% a compostos farmacêuticos.

**Conclusión.:**

A tendência mundial é voltada para patentes de tratamento de complicações do pé relacionadas ao diabetes.

## Introduction

Diabetic foot ulcers (DFU) is defined by the presence of infection, ulceration, and/or destruction of deep tissues, usually associated with peripheral neuropathy and vascular impairment in people with diabetes mellitus (DM).^1^ Among the complications, DFU is among the most serious and affect the person's quality of life.^2^ The combination of ischemia, loss of protective sensation, and mechanical factors favors the emergence of chronic lesions in the lower limbs. Without timely and adequate clinical intervention, these lesions present a high risk of progression to severe infection and partial or total amputation of the affected limb.^1^ DM is considered one of the chronic non-communicable diseases with the greatest prospect of increasing numbers in the coming years.^2^ In this context, technological progress emerges as a possibility to address historically complex and seemingly unsolvable clinical challenges. An important driver of the dissemination of health technologies is the patent system. In addition to guaranteeing incentives and protection for researchers and stimulating the development of the health sector, they are considered important indicators of the level of technological advancement for the capacity to transform technical-scientific knowledge into economically viable solutions.^3^ However, developing countries face significant difficulties in accessing technologies, including economic, cultural and structural barriers.^4^ Thus, the analysis of patented technologies offers strategic support for identifying emerging solutions that contribute to the qualification of health care,^4^ while highlighting the predominant directions of technological innovation in specific areas. Therefore, systematic studies on patents focused on the evaluation, prevention and treatment of foot ulcers in people with diabetes are justified, given their clinical, social and technological relevance.

## Methods

This is a technological foresight study, characterized as a tool for mapping scientific technologies to generate future predictions used in broad perspectives, such as industrial, economic, social, and health-related contexts, through planning and management tools.^5^ The guidelines of the Joanna Briggs Institute (JBI)^6^ and the Preferred Reporting Items for Systematic Reviews and Meta-Analyses extension for Scoping Reviews (PRISMA-ScR) checklist^7^ were used to conduct the research. The guiding question of this study was developed using the mnemonic strategy to describe the participants, concept, and context (PCC). P (Population) represents “people with diabetes”; C (Concept), “patent records”; and C (Context), “available technologies related to diabetic foot ulcers.” Thus, the following research question we formulated: what is the trend of the available international patent records related to diabetic foot ulcers?

This study used the World Intellectual Property Organization (WIPO) patent database for data extraction. The choice of this platform is justified by its free access and by the significant number of patent documents filed worldwide,^8^ allowing researchers, investors, and companies to learn about developed innovations. The descriptor “diabetic foot,” included in the Health Sciences Descriptors (DeCS), was used to search the platform.

Data collection was limited to the last five years (2020 to 2024). The process was conducted over six months (May to December 2024). For data collection on the WIPO platform, the following electronic address was accessed: [https://patentscope.wipo.int/search/en/search.jsf](https://patentscope.wipo.int/search/en/search.jsf). In the “PATENTSCOPE Simple Search” tab, there are two fields: “Field” and “Search terms.” In the first field, “Front Page” was selected, and in the latter, the English descriptor “Diabetic Foot” was used. After performing the search, a new tab containing the results and filter options was displayed. A filter was applied to the initial search results by clicking on a data-bar-shaped icon called “Analysis.” In “Analysis,” five categories are presented: “Countries,” “Applicants,” “Inventors,” “IPC code,” and “Publication Dates.” A new filtering step was then carried out by selecting “Publication Dates.” Example: “Front Page” > “Diabetic Foot” > “Analysis” > “Publication Dates” > “2024.” Inclusion criteria were complete records that addressed the research question. Exclusion criteria were technologies related to diabetes complications not involving the feet, equipment for transporting medical supplies, and material storage devices. The initial screening of patents was performed by reading the titles and abstracts of the registered patents found. The records selected based on the inclusion and exclusion criteria were analyzed in full. Those that addressed the research question proceeded to the extraction of the following variables: country of origin, patent registration code, applicants, filing and publication dates, as well as variables related to the functionality and applicability of the patents. The database was organized using Excel® 2024 software.

Data analysis was based on the theoretical concepts presented in the “Practical Guidelines on the Prevention and Management of Diabetes-Related Foot Disease,” developed and updated by the International Working Group on the Diabetic Foot (IWGDF). The IWGDF is an international organization that develops evidence-based guidelines for the prevention and treatment of diabetes-related foot diseases. It is composed of specialists from various fields and aims to reduce the impact of diabetic foot disease by publishing updated practical guidelines every four years, including recommendations, criteria, and definitions that are widely used in clinical practice and research.^2^

### Results

The initial search resulted in the identification of 1426 patent records after reading titles and abstracts. In the analysis of the full records, 54 records were excluded for these reasons: technologies focused on non-foot-related diabetes complications (*n*=41), medical supplies transport equipment (*n*=4) and packaging devices (*n*=9). A final sample of 1372 patents was obtained. With an annual mean of 275 published records, the mean time between the filing date and publication was 278 days, with little variation across the years analyzed (between eight and ten months), revealing delays in this process.

The geographic origin of the records showed a predominance of China as the main applicant, accounting for 76.38% of all records related to diabetes-associated foot ulcers. Next were the United States (4.74%), the Patent Cooperation Treaty (3.57%), India (3.13%), and the European Patent Office (2.55%). Other countries with relevant contributions included Russia (1.68%), Mexico (1.60%), South Korea (0.95%), Canada (0.87%), Australia (0.80%), Japan (0.73%), and New Zealand (0.51%). The remaining records originated from countries such as the Philippines, Denmark, Malaysia, Poland, Bulgaria, the United Kingdom, Jordan, Tunisia, Thailand, Spain, Vietnam, Singapore, Indonesia, Costa Rica, and Georgia. No patent records of Brazilian origin were found in this study.

Most records correspond to medical devices (999; 72.81%), while 27.19% (n = 373) are pharmaceutical compositions. Applicants such as the United States, the Patent Cooperation Treaty, the European Patent Office, Mexico, Canada, Japan, and New Zealand concentrate their records on pharmacological solutions. In contrast, countries like China, India, Russia, South Korea, and Australia stand out for their substantial volume of records focused on medical devices. The patents were classified into three groups: (i) assessment; (ii) prevention; and (iii) treatment. Most records focus exclusively on therapeutic interventions, highlighting the development of solutions aimed at treating already-established complications rather than innovations related to the assessment and prevention of diabetes-associated foot ulcers. China stood out as the leading applicant country across all these categories (Table 1).

**Table 1:** Categorization of 1372 patent records on diabetic foot ulcers according to their applicability and origin

Origin of depositors	**Assessment (*n*=78)**	**Prevention (*n*=167)**	**Treatment (*n*=1127)**	Total (%)
China	34	136	878	1048 (76.38)
USA	11	6	48	65 (4.74)
Patent Cooperation Treaty	5	4	40	49 (6.57)
India	10	7	26	43 (3.13)
European Patent Office	4	4	27	35 (2.55)
Russia	2	3	18	23 (1.68)
Mexico	1	0	21	22 (1.60)
South Korea	5	0	8	13 (0.95)
Canada	1	0	11	12 (0,87)
Australia	3	1	7	11 (0.80)
Japan	0	2	8	10 (0.73)
New Zealand	0	1	6	7 (0.51)
Other	2	3	29	34 (2.48)

The analysis of 78 patent records focused on the assessment of diabetic foot ulcers found that most refer to ulcer classification (66.7%), followed by those aimed at identifying associated comorbidities (17.0%) and innovations for determining the cause of the lesion (15.4%). Regarding ulcer classification, the technologies present innovations that operate through analysis and prediction systems based on the fusion of multiple data. With respect to the identification of comorbidities, the records included, for example, systems for risk assessment based on multiple physiological parameters and portable devices for screening and monitoring. Concerning the determination of the cause of the ulcer, there are records of diabetic foot detection methods based on image analysis. Among the 167 records related to ulcer prevention, most correspond to examination devices (50.3%), followed by innovations concerning the evaluation of risk factors and pre-ulceration signs (38.9%) and records on health education (10.8%).

A predominance of patents focused on examinations was observed, approximately 4.8 times higher than the number of technologies aimed at health education. Among the examination devices, this study included those involving measurement

of susceptibility to diabetic foot ulcers using physical biomarkers, sampling devices and clinical detection for diabetic foot, and devices and methods for testing pressure thresholds in diabetic foot. Records related to the evaluation of risk factors and pre-ulceration signs were primarily associated with tissue pressure and foot perfusion. Devices were found operating through prediction methods for the development of severe distal neuropathy and diabetic foot syndrome, the application of biochemical markers predictive of ulcers, orthoses, and methods for callus removal, massage devices, and devices for heat preservation in the soles of the feet. Regarding innovations related to health education, this study highlighted a Chinese proposal for an aid box for teaching about experiences with diabetic complications, a training device for dressing changes, and an intelligent teaching model.

In the group of patents aimed at the treatment of diabetes-related foot ulcers, there was a predominance of technologies directed at wound cleansing, followed by alternative therapies outside the framework used for classification in this study. There were also a considerable number of records related to pressure redistribution; drugs for infections; alternative therapies, according to the theoretical framework used in this study; and ulcer protection. Other patent records related to tissue reperfusion, surgical intervention technologies, rehabilitation devices, and Peripheral Arterial Disease (PAD) assessment (Table 2).

**Table 2:** Records of 1127 patents on the treatment of diabetic foot ulcers

Treatment	Records	%
Assessment of peripheral arterial disease	6	0.53
Rehabilitation devices	15	1.33
Surgical intervention technology	17	1.51
Tissue reperfusion	33	2.93
Ulcer protection	52	4.61
Alternative therapies 1*	59	5.24
Infection medication	70	6.21
Pressure redistribution	167	14.55
Alternative therapies 2**	323	28.66
Wound cleansing	388	34.43

Notes: *Technologies that are in accordance with the IWGDF theoretical framework

**Technologies that are not in accordance with the IWGDF theoretical framework.

When we analyzed the records related to wound cleansing, instruments for debridement, antiseptic solutions, surfactant agents, dressings, and especially foot supports that optimize dressing changes were found. The “alternative therapies 2” category included records of enzymatic and glucose-based dressings, a lipocalin-2 inhibitor, impedance-based l-tyrosine detection bandages, a healing lotion based on *Ganoderma lucidum*, miRNA-146 application, stem cell applications, and super-expressed miR-13474 exosome applications. Pressure redistribution included patent records for compression stockings, insoles, customized shoes, foot supports, and cushions. Regarding infection-related pharmaceuticals, notable items included formulations and production methods such as antibacterial hydrogels, linezolid-based medications, mesenchymal stem cells as antibacterial agents against *Pseudomonas aeruginosa*, and piperidinyl-tetrahydroquinolines used as alpha-2C adrenoceptor antagonists.

The patents classified as “alternative therapies 1” referred to treatment approaches not covered in the other categories but aligned with the theoretical framework of this study, such as a membrane composed of platelet-rich fibrous protein, as well as the method for preparing and applying this protein, and the process for obtaining a semisolid primary platelet-rich fibrin dressing. Tissue spacers or layered devices for edema protection and pneumatic insulators were technologies identified for ulcer protection. Patents related to tissue reperfusion included thermal maintenance equipment, massage instruments, and devices for treatment and rehabilitation of diabetic feet based on a circulation system and dual lower-limb auxiliary control. Records of surgical intervention technologies presented methods and instruments for tissue repair, in-hospital debridement, and amputation. Rehabilitation devices accounted for the second lowest number of patents related to treatment modalities. The filings included physical rehabilitation devices that reduce restrictions caused by ulcers and physiotherapy equipment.

Considering Peripheral Arterial Disease (PAD) assessment, devices and methods were found for monitoring peripheral diabetic neuropathy, as well as a photoacoustic spectrum method based on quantitative assessment of diabetic foot vasculopathy.

## Discussion

This study reveals that countries such as China, the United States, and India have emerged as the largest filers of patents aimed at the evaluation, prevention, and treatment of diabetes-related foot ulcers. A large share of these patents is registered through the Patent Cooperation Treaty (PCT) and the European Patent Office (EPO). Data from the 2024 Global Innovation Index corroborate these findings, highlighting China in 11th place in the overall ranking and first among upper-middle-income countries in the classification of innovative economies by income group. The United States appears in third place in both the overall ranking and the high-income subset, whereas India ranks 39th, leading among lower-middle-income countries.^8^ The PCT is an international treaty administered by the World Intellectual Property Organization (WIPO), comprising 158 States, which allows a single patent application to be filed in order to seek protection in several countries simultaneously. The European Patent Office (EPO) is an organization that enables inventors to obtain patent protection for their inventions in Europe through a single process and in the same place. Centralized offices and international treaties use patent- processing acceleration agreements and play an important role in promoting high-quality innovation in international trade by reducing assessment uncertainty. As a result, companies are encouraged to increase the quality of exports in patent- intensive sectors, leading to differentiated products.^9^

The number of patents related to medical devices was significantly higher compared to those aimed at pharmaceutical compounds. This occurs because the clinical development of new drugs is highly limited, as only a small fraction manages to advance to regulatory review. In the United States, issues such as ineffective results with respect to the main study objectives or the presence of severe adverse reactions lead to the early termination of most late-phase trials. Only about 10% of molecules under clinical evaluation reach the submission stage at the Food and Drug Administration (FDA).^10^ The patent analysis reveals a predominance of patents focused on treatment, showing a significantly larger number compared to the evaluation and prevention groups. This reflects the persistent predominance of the biomedical model-albeit inefficient-in the way care models are delineated.^11^ In the group of patents classified as evaluation, there is a predominance of innovations focused on classification. Increasing importance has been given to early detection and rapid intervention to prevent complications and reduce morbidity related to diabetic foot.(1) The classification of a Diabetic Foot Ulcer is a complex procedure conditioned by multiple independent factors that influence the severity of the lesion.^12^

In this context, assessment tools such as the SINBAD scoring system -an acronym representing six variables: site, ischemia, neuropathy, bacterial infection, area, and depth-^12^ gain relevance, as they are easy to apply, do not require specialized devices, and rely solely on data obtained during clinical examination, offering important support for triage performed by qualified professionals.^13^ Currently, systems are being developed for the analysis and prediction of diabetic foot based on the integration of different types of data. A recent study employed multimodal deep-learning techniques to predict the risk of diabetic foot in patients with type 2 DM, combining tongue images with clinical information. The model showed high performance in terms of accuracy and sensitivity, demonstrating the potential of fusing qualitative and quantitative data.^14^ In addition, emphasis has been placed on the use of imaging devices that contribute to ulcer classification, especially those related to diabetic foot, through imaging-capture technologies combined with machine- learning algorithms. Research highlights the application of computer-vision and artificial intelligence techniques in the detection, analysis, and monitoring of these lesions. Such approaches enable remote evaluation of wound images, promoting significant advances in wound care by allowing patients themselves to monitor their progress and enabling health professionals to perform remote triage.^15^

Although the IWGDF Guidelines^2^ state that identifying comorbidities and determining the cause are crucial in the DFU assessment phase, few innovations have been recorded focusing on this important aspect. This scenario highlights a stagnation in the technological advancement of the area, largely attributed to the lack of integration between basic and clinical research. Added to this limitation is the non-standardized collection of clinical and biological data, which compromises the quality and reproducibility of the evidence.^16^ Furthermore, the high cost of developing new technologies represents an

additional obstacle. However, this challenge can be overcome with the use of accessible and low-cost solutions, such as portable imaging devices based on optical principles and other simple techniques capable of evaluating multiple physiological parameters directly at the bedside.^17^

In the identification of comorbidities, devices that assess multiple physiological parameters are being explored, combined with portable technologies aimed at screening and monitoring metabolic conditions. One example is the portable biochip attached to smartphones, capable of non-invasively monitoring glucose and insulin levels in saliva, showing promise in the early detection of changes consistent with pre-diabetes and diabetes.^18^ In the group of prevention of diabetic foot ulcers, examination devices stood out. They assist in the early detection, monitoring, and measurement of ulcer susceptibility.^2^ They allow, through physical biomarkers such as pressure and temperature, the early identification of changes in tissue blood flow, revealing an imminent ulceration.^19^ This predominance again reveals the biomedical model of health care, which emphasizes the diagnosis and treatment of diseases instead of preventive and promotional measures. Furthermore, there is a trend towards patent registrations of high technological density, which may be because it is more lucrative compared to preventive technologies.

Patents on the assessment of risk factors and signs of pre-ulceration were also significant in this group. Controlling plantar temperature and pressure using smart technologies, as demonstrated by a comparative clinical study of three insole models, is effective in assessing and correcting biomechanical risk factors.^20^ This clinical research concluded that modifications to the surface of the insoles reduce temperature and pressure points in the foot area, showing positive effects in preventing foot complications.^20^ This fact reaffirms the advantage of monitoring and controlling risk factors, characterizing it as an essential preventive tool in patient care, which validates the development of these technologies that offer better adherence to treatment.

However, we observed a limited number of health education technologies. Professional education tools allow the development of professional and teamwork skills and competencies. At the same time, they contribute to patient safety.^21^ Nevertheless, one explanation for the low investment may be related to the indirect and long-term return on capital invested in the development of educational technologies, when compared to examination devices, which have a highly profitable market value. It is also noteworthy that the group of records related to the treatment of diabetic foot ulcers stood out. The analysis of patent records shows significant concern with the initial stages of care for diabetic foot ulcers, especially regarding wound bed preparation and the control of pre-existing infections. The substantial volume of technological innovations in the “ulcer cleansing” subgroup reinforces this premise by demonstrating the relevance of technologies aimed at mechanical cleaning and tissue decontamination of the lesion. Adequate wound bed cleansing, through mechanical methods and appropriate devices, promotes healing progression and substantially reduces the risk of local infection.^22^ Consequently, a smaller proportion of patents related to topical drugs or antimicrobial dressings is observed, which may indicate a preference for approaches that prioritize the physical management of the wound rather than pharmacological therapies for biofilm eradication in already infected lesions.

Alternative therapies not aligned with the theoretical framework used in this study^2^ highlight the importance of constantly updating protocols, guidelines, and consensus documents in the face of technological advances in healthcare. These documents are the fundamental scientific framework for guiding professionals by establishing quality standards, procedures, and actions to improve healthcare.^23^ It is through evidence-based practice (EBP) that professionals should seek to enhance their knowledge and apply it in their workplace. Tissue reperfusion, pressure redistribution, and ulcer protection technologies, in turn, are examples of continuous-use resources, such as compression stockings, insoles, adapted footwear, and massagers, which aim to improve tissue oxygenation, provide plantar offloading, and protect against mechanical trauma. The purpose of these technologies is to reduce biomechanical factors associated with the risk of ulceration.^2^ However, these treatment inventions require patients’ adherence for their effectiveness; therefore, technologies aimed at structured patient education are important so that they understand the importance and necessity of the prescribed treatment.

Surgical intervention technologies were also observed, revealing the clinical severity of patients who will require surgical debridement or limb amputation. The presence of such invasive intervention highlights situations in which preventive and treatment measures implemented by healthcare professionals were not effective in preventing damage, resulting in limb

loss. It is also emphasized that patient engagement with treatment influences either the improvement or worsening of their clinical condition and its possible outcomes. Rehabilitation devices are also considered important interventions recommended by the IWGDF Guidelines,^2^ but the patent findings in this study were limited. Pressure offloading devices are an effective intervention in the recovery of people with diabetic foot ulcers.^24^ In addition to enabling better treatment adherence by patients, rehabilitation devices minimize mechanical stress and shear, which supports wound recovery and patient autonomy. Therefore, these inventions are essential for the rehabilitation of patients resistant to adhering to care proposals.

Finally, despite the wide range of these healing technologies, there is a deficit in inventions related to technological devices that assist in the assessment of PAD. The identification of PAD aids in the treatment of diabetic foot ulcers, requiring urgent treatment,^2^ since early intervention minimizes major complications. The use of photoacoustic spectral methods and imaging investigation allows for better assessment of the microcirculatory system due to its high sensitivity. A clinical study, using NIR- II imaging, emphasizes as a positive result the specificity and sensitivity of the device that allows for the non-invasive assessment of tissue perfusion capacity of the limbs.^25^ Therefore, investment in highly specific and sensitive technologies that allow for the assessment, identification, and detection of perfusion alterations and other factors capable of corroborating the imminent onset of foot ulcers in people with diabetes becomes fundamental. Investment in research that makes it possible to reduce the complications and aggravations associated with the condition of people with diabetes is essential, considering the growing trend of chronic non-communicable diseases worldwide.

Conclusion. The analysis of patents related to diabetic foot ulcers reflects a significant number of international technological solutions primarily aimed at treating complications. Despite their relevance for early intervention and minimizing complications, the areas of prevention and assessment lack technological innovation, creating opportunities for advancing technological development research. The articulation between investments in applied research, knowledge dissemination, international cooperation, and political commitment can bring advances towards safety, improved quality of life, and person-centered care for people with diabetes.

Limitations of the study. The topic deserves to be explored in other databases and with an extended data collection period. We can also mention the lack of analysis of unpublished documents due to the confidentiality period of patent offices.
